# Microstructural Characteristics of Al-Ti-B Inoculation Wires and Their Addition to the AlSi7Mg0.3 Alloy

**DOI:** 10.3390/ma15217626

**Published:** 2022-10-30

**Authors:** Anna Knaislová, Štefan Michna, Iryna Hren, Tomáš Vlach, Alena Michalcová, Pavel Novák, Dana Stančeková

**Affiliations:** 1 Faculty of Mechanical Engineering, Jan Evangelista Purkyně University in Ústi nad Labem, Pasteurova 3334/7, 400 01 Usti nad Labem, Czech Republic; 2 Department of Metals and Corrosion Engineering, University of Chemistry and Technology, Technická 5, 166 28 Prague, Czech Republic; 3 Department of Machining and Production Technologies, Faculty of Mechanical Engineering, University of Zilina, 010 26 Zilina, Slovakia

**Keywords:** inoculation, Al-Ti-B, AlSi7Mg0.3, grain size, intermetallics

## Abstract

Commercially supplied inoculation wires have a guaranteed chemical composition but not the size and distribution of individual phases, which are very important for nucleation. Therefore, two commercial alloys used for the inoculation of Al-Si alloys (AlTi3B1 and AlTi5B1) are investigated in this paper. The emphasis is placed on their structural analysis and the size and distribution of individual intermetallic phases. Furthermore, the grain refinement effect will be tested by adding these alloys to the AlSi7Mg0.3 alloy and testing the optimal amount of added inoculation wires. The results showed that the size and distribution of the individual phases in AlTi3B1 and AlTi5B1 meet the requirements for the successful inoculation of aluminum alloys, the intermetallic phases based on the TiAl_3_ phase are fine enough, and there is no agglomeration that would reduce the number of nuclei. This assumption was confirmed by adding these inoculants to the AlSi7Mg0.3 alloy, and it was found that the most ideal amount of inoculants added is 0.01 wt % when the structure was refined by approximately 32%.

## 1. Introduction

The refinement of the grain of aluminum alloys can be achieved by inoculation [1,2,3]. Inoculation affects the total number of nuclei and therefore results in a refinement of the structure. For aluminum, inoculation is used to refine the α-phase of the metal matrix, that is, the solid solution by adding some transition metals [4].

Inoculation is particularly effective for Al-Si alloys with a high proportion of α-solid solution in the structure, that is, Al-Si alloys with a silicon content in the range of 1–12 wt % [2,5,6,7]. The refinement of the solid solution is carried out by adding the elements titanium and boron, which are added to the melt individually or in combination [8,9]. These elements are added to the melt in the form of intermetallic compounds, which are contained in the master alloys. Titanium is added to the melt by adding master alloys (e.g., AlTi_6_) that contain the intermetallic compound TiAl_3_ [10]. Boron is added to the melt by adding master Al-B-type master alloys (e.g., AlB_4_) that contain the intermetallic compound AlB_2_ [11,12]. Titanium and boron in combination with each other are added to the melt by adding master alloys (e.g., AlTi5B1, AlTi5B0.2), which contain elements Ti and B in the form of intermetallic compounds TiB_2_ and TiAl_3_. The intermetallic compound TiB_2_ is insoluble in α-solid solution, while the compounds AlB_2_ and TiAl_3_ are soluble in α-solid solution [13,14,15,16].

Inoculation with titanium and boron gives satisfactory results in α-solid solution refinement. However, these elements have a weaker softening effect than boron separately but a higher softening effect than titanium. The refinement of the α-solid solution with titanium and boron in combination is carried out by the action of the phases TiAl_3_ and TiB_2_, (TiV)B_2_, AlB_2_, AlB_12_ or AlTiB, which are added to the melt by master alloys [17,18,19,20]. Vanadium may be present in the metal resulting from electrolysis and may react with elements of the grain refiner to form (TiV)B_2_ [21]. This particle is similar to TiB_2_ but larger in size. These master alloys are produced with different titanium and boron contents. The most commonly used master alloys have the titanium and boron contents of 5% and 1%, respectively [22]. This ratio has been proven to be the most advantageous in practice, as the efficiency of inoculation decreases as this ratio is further increased. Boron is completely bound to the insoluble TiB_2_ phase, which is usually very finely dispersed in the master alloy. The rest of the titanium content (approximately 2.8 wt %) precipitates in the form of polyhedral particles of the melt-soluble TiAl_3_ intermetallic phase [13,14,19]. This is in contrast to the Al-Ti binary diagram, which shows that at real melting temperatures of aluminum alloys, the TiAl_3_ solubility of the intermetallic phase is almost negligible [23].

**Figure 1:**
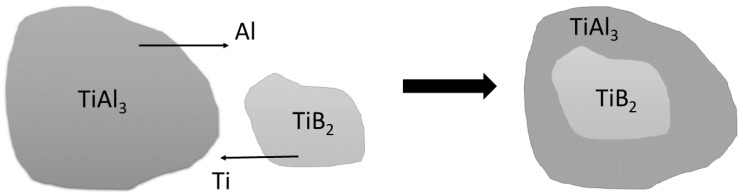
Graphical representation of the dissolution of TiAl_3_ intermetallic phases.

Boron is found only in the insoluble intermetallic phase TiB_2_, which is usually very finely precipitated in the master alloy. The remainder of the titanium content (approximately 2.8 wt %) is precipitated in the form of polyhedral particles of the intermetallic phase TiAl_3_. TiB_2_ particles in the TiAl_3_ phase have a crystal lattice similar to aluminum alloys and cannot become a nucleus. The diffusion of aluminum in the TiB_2_ phase and the diffusion of titanium from the TiB_2_ phase form a shell on these particles, which is composed of the TiAl_3_ phase. Therefore, it results in the particles formed by the nucleus of the TiB_2_ phase and the TiAl_3_ shell. These particles are potential nuclei. The remaining completely undissolved particles of the TiAl_3_ intermetallic phase of the master alloy also act as nuclei, although they are not commonly identified in castings [24]. The dissolution of the intermetallic phase TiAl_3_ is shown graphically in Figure 1.

The addition of Al-Ti or Al-Ti-B-based inoculants to Al-Si alloys is used industrially. However, industrial companies are not interested in the microstructure of inoculant alloys, only their chemical composition and their effect on the grain refinement of the Al-Si alloy [8,9,25,26,27,28,29,30,31]. The primary task of the inoculants added to the Al-Si alloy is to refine the microstructure by heterogeneous nucleation and to control grain growth [2]. The grain refiners (inoculants) in Al-Si alloys focus on suppressing the formation of coarse columnar grains and support the formation of finer equiaxed grain structures during solidification [2]. An important criterion for the structural description of aluminum alloys is the distance between the secondary dendrite axes, which is known as SDAS (Secondary Dendrite Arm Spacing). The smaller the size of the primary grains and the distance between the secondary axes (SDAS), the better the chemical and structural homogeneity of the alloy and the better the mechanical properties of the alloy. A number of other structural phenomena are also related to the size of the SDAS. The appearance of a finer structure (i.e., with a smaller SDAS value) is associated with smaller segregation distances and with a smaller extent of segregation, therefore, smaller particles of intermetallic inclusions are formed. Impurities are separated as separate particles in the interdendritic spaces and do not form a network; the chemical composition of the alloy is more homogeneous, and the microporosity is more favorably distributed. Therefore, the smaller the SDAS value, the higher the mechanical properties of the casting.

McCartney created the first idea of the effect of titanium and titanium with boron to refine the aluminum grain. At the same time, he found that boron in combination with titanium is more suitable than titanium with carbon [25]. Spittle [26] investigated the addition of the AlTi5B1 alloy to Al-Si alloys with a very small amount of silicon investigated by Spittle [26]. Mohanty found that grain refinement is caused not only by TiB_2_ titanium boride but also by TiAl_3_ titanium aluminide [27]. Al-Si alloys with silicon content greater than 7 wt % were studied by Sritharan [9], who found that a more powerful inoculant should have the same ratio of titanium and boron [9]. Kumar, on the other hand, found that the carbon inoculant had a better effect than the boron inoculant [28]. The solution could be to add titanium, boron and carbon to the inoculant at the same time, as performed by the researchers in [29]. Jia [32] investigated the addition of Al-Ti-B-Y grafts to the Al-Si-Mg alloy.

The aim of this paper is to characterize two commercially used inoculant alloys with the addition of titanium and boron that are used to refine a solid solution of Al-Si alloys. The AlTi3B1 and AlTi5B1 inoculation wires were characterized in terms of phase composition, microstructure, grain size, and microhardness. Furthermore, their effect of refining the solid solution was tested by adding these inoculants to the AlSi7Mg0.3 alloy, and the optimal amount of inoculants was determined.

## 2. Materials and Methods

In this experiment, two Al-Ti-B alloys that differ in titanium content were studied. Both materials were supplied by Foseco (Foseco, Vesuvius Group Foundry Technologies Division, Tamworth, UK) in the form of master alloys for inoculation of aluminum alloys. The supplied wires were cut, and metallographic samples were prepared from the wires. The samples were mounted in Varidur 200 (Buehler, Braunschweig, Germany) methacrylate resin, ground on P80 to P4000 sandpapers (Hermes Schleifmittel GmbH, Hamburg, Germany) and polished by Eposil F suspension mixed with hydrogen peroxide (ratio 1:5). Subsequently, polished samples were etched using modified Kroll’s reagent (10 mL HF, 5 mL HNO_3_, and 50 mL H_2_O) prepared in the laboratory.

Phase identification was performed by X-ray diffraction analysis using a X’Pert Pro diffractometer (PANalytical, Almelo, The Netherlands), which was followed by evaluation in the X’Pert HighScore 3.0 software package (PANalytical, Almelo, The Netherlands) using the PDF-2 2018 database.

The inoculation wires were cut to small cylindrical samples having the height of 0.8 mm and ground on P80 do P1200 sandpapers. The microstructure of the inoculation wires was investigated using a LEXT OLS 5000 laser confocal microscope (Olympus, Sindzuku, Japan) and a TESCAN VEGA 3XMU scanning electron microscope (TESCAN, Brno, Czech Republic) with an Oxford Instruments X-max 20 mm^2^ EDS (Energy-dispersive X-ray spectrometer) analyzer (Oxford Instruments, HighWycombe, UK). The particle size was determined from the micrographs by the means of image analysis using the analysis application software of Olympus OLS 5000.

Both inoculation wires were added to the AlSi7Mg0.3 alloy (i.e., the alloy containing 92.7 wt % aluminum, 7 wt % silicon and 0.3 wt % magnesium). The AlSi7Mg0.3 alloy was melted at 760 °C in a graphite crucible. The samples were cast by gravity casting in a mold preheated at 200 °C; the molds had a conical shape with a height of 700 mm, a top diameter of 850 mm and a bottom diameter of 400 mm. The AlSi7Mg0.3 alloy was chosen to optimize the amount of the inoculant wire. The composition of the alloys is shown in Table 1.

The AlTi5B1 inoculant was added as wire at the concentration of 0.01 wt %, 0.05 wt %, 0.1 wt % and 0.2 wt %, and the exposure time of the inoculant in the melt was 6–7 min. Subsequently, the selected amount of inoculant was also used for the addition of AlTi3B1 to AlSi7Mg0.3. For comparison, the AlSi7Mg0.3 alloy without inoculants was cast in the same way. All five melts were processed under the same conditions (temperature, time). Samples were taken from the same place: namely, the intercentral region, for macroscopic and microscopic evaluation of the size of dendritic cells (α-solid solution). All alloys were mounted to the resin, ground, and polished. Their microstructure was investigated using a laser-confocal microscope.

**Figure 2:**
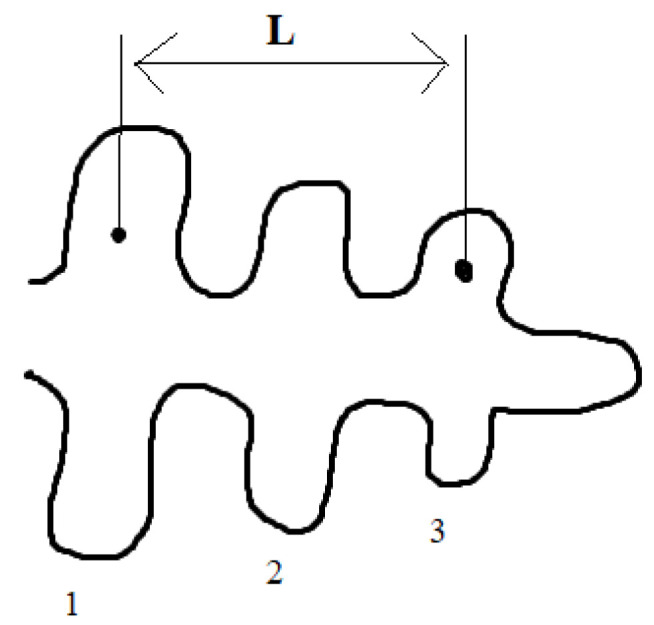
SDAS measurement scheme: L is the distance of secondary arms; 1, 2 and 3 are the axes.

The most common method to determine the dendritic structure is the so-called *SDAS* (Secondary Dendrite Arm Spacing) method (Figure 2). *SDAS* is the distance between the secondary axes of the dendrites. On the selected dendrite, the distance across several secondary arms (more than two) was measured on the metallographic sample and divided by the number of gaps; see Equation (1):

(1) $$SDAS=\frac{L}{n-1}$$

where *L* is the distance of the secondary arms and *n* is the number of axes.

## 3. Results

### 3.1. Characteristics of the Inoculants

**Figure 3:**
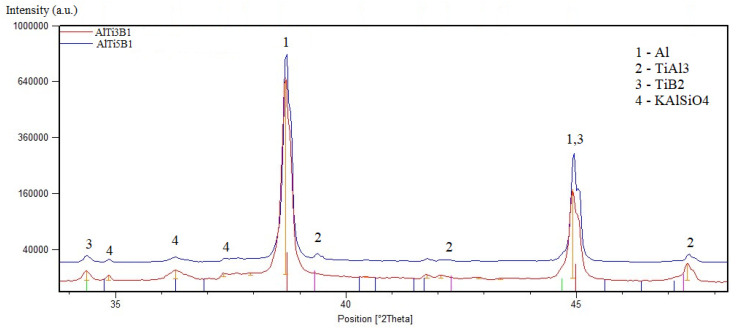
XRD patterns of AlTi3B1 and AlTi5B1 alloys.

The phase identification of both alloys determined by X-ray diffraction is shown in Figure 3. The AlTi3B1 and AlTi5B1 alloys have the same phase composition, consisting of aluminum (fcc structure), titanium aluminide TiAl_3_ (hcp structure), and titanium diboride TiB_2_ (hcp structure). In XRD patterns, there is also the KAlSiO_4_ phase (trigonal structure), which is from the embedding material.

**Figure 4:**
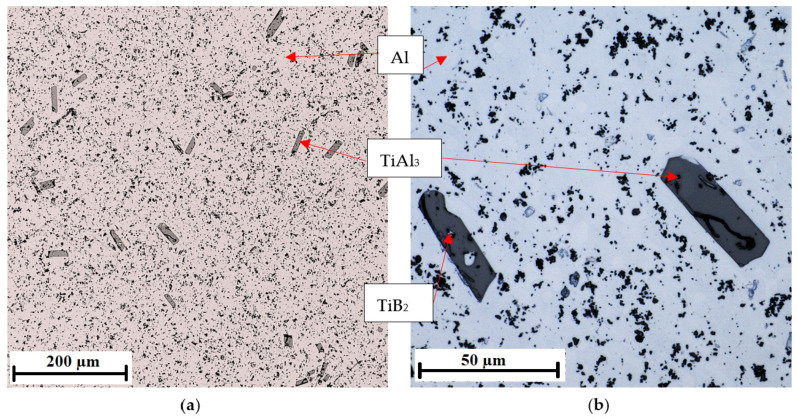
Microstructure of the AlTi3B1 alloy with coarse-edged TiAl_3_ intermetallic phases: (**a**) magnification 5×, (**b**) magnification 20×.

The microstructure of the AlTi3B1 inoculation wire shows an aluminum matrix, large elongated titanium aluminide that are randomly oriented, and fine flaky irregular-shaped particles (Figure 4). The coarse sharp-edged intermetallic phases are TiAl_3_ titanium aluminide particles. Inside the coarse intermetallic phases of TiAl_3_, fine irregular particles of TiB_2_ are visible (Figure 4b).

**Figure 5:**
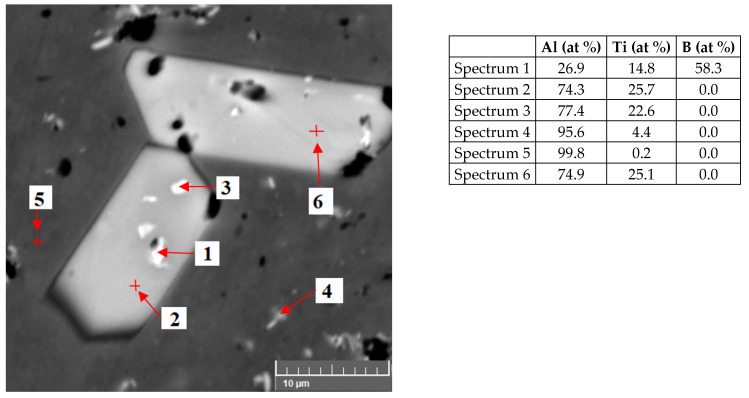
Microstructure of the AlTi3B1 alloy and EDS point analysis.

The microstructure of the AlTi3B1 alloy from the scanning electron microscope in Figure 5 shows a coarse plate-shaped particle, where the point EDS analysis of this particle confirms the intermetallic phase TiAl_3_ (spectrum 2, Figure 5). Scanning electron microscope images show that fine irregular particles of lighter color are visible inside the intermetallic phases of TiAl_3_ (Figure 5). EDS analysis of this particle confirms that it is an intermetallic phase of TiB_2_ (Spectrum 1, Figure 5). The matrix consists of aluminum (spectrum 5, Figure 5).

**Figure 6:**
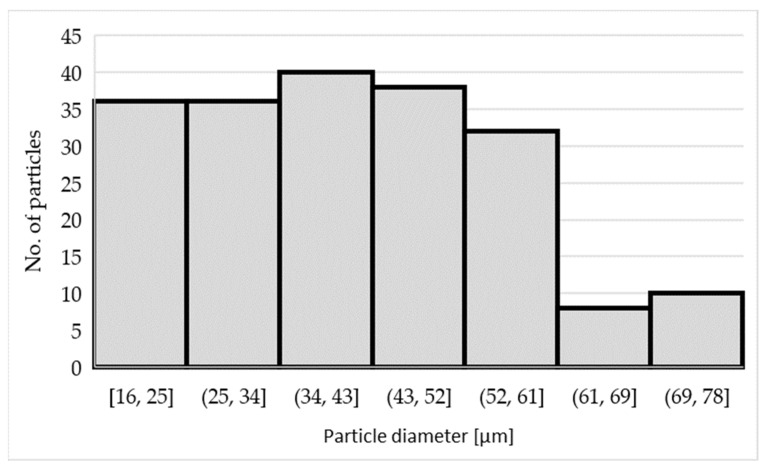
Histogram of the particle size distribution of the TiAl_3_ phase in the AlTi3B1 alloy.

The microstructure of the laser confocal microscope (Figure 4) shows large elongated particles TiAl_3_ of size 37 ± 17 µm and fine flaky particles of irregular shape TiB_2_ of size 0.5–2 µm. The histogram of the particle size distribution of the TiAl_3_ phase is shown in Figure 6. These values were obtained from the image analysis (images from the laser confocal microscope) using analysis application software. More than 200 particles were included in the analysis. In the sample, there is approximately the same amount of particles with a particle diameter of 16 to 61 µm.

**Figure 7:**
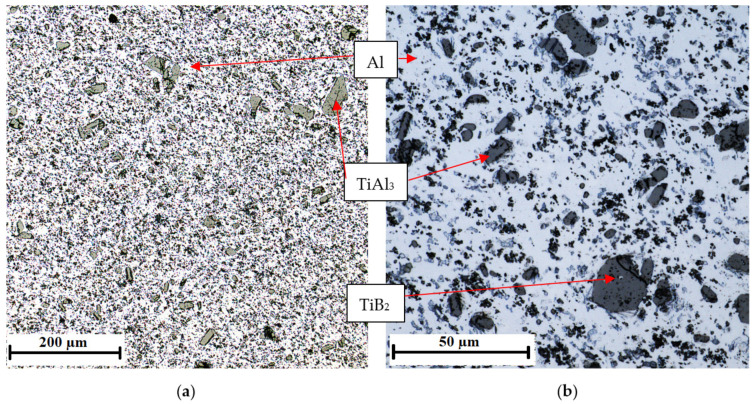
Microstructure of the AlTi5B1 alloy with coarse-edged TiAl_3_ intermetallic phases: (**a**) magnification 5×, (**b**) magnification 20×.

The microstructure of the AlTi5B1 inoculation wire shows the aluminum matrix and large plate-shaped or coarse-edged or irregularly shaped intermetallic particles and fine flaky particles of irregular shape (Figure 7). Compared to the AlTi3B1 alloy, the AlTi5B1 alloy has a larger number of TiAl_3_ intermetallic phases in the structure with different irregular shapes.

**Figure 8:**
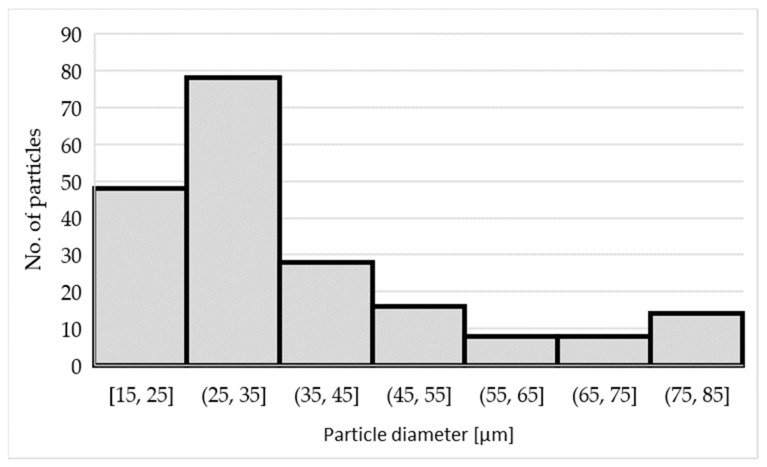
Histogram of the particle size distribution of the TiAl_3_ phase in the AlTi5B1 alloy.

The TiAl_3_ intermetallic phase is present in the AlTi5B1 inoculation wire as quite coarse particles with a size of 41 ± 15 µm. The histogram of the TiAl_3_ phase particle size distribution is shown in Figure 8. 

**Figure 9:**
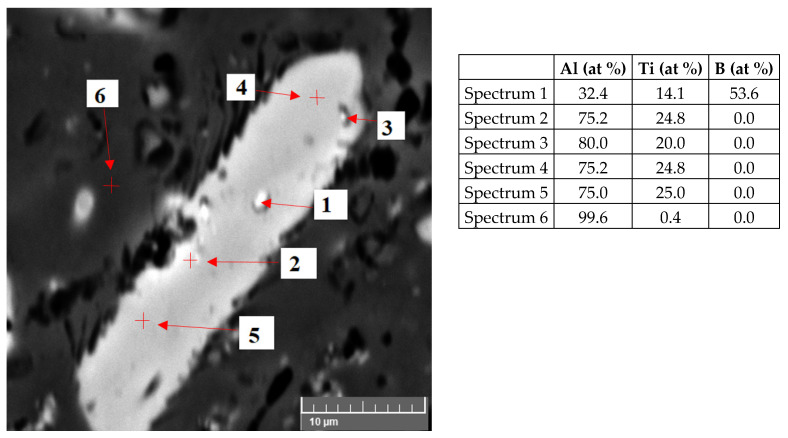
Microstructure of the AlTi5B1 alloy and EDS point analysis.

The scanning electron micrograph of the AlTi5B1 alloy in Figure 9 shows a coarse plate-shaped particle, where the point EDS analysis confirms that it is the TiAl_3_ intermetallic phase (spectrum 2, Figure 9). Within this coarse intermetallic phase of TiAl_3_, a fine irregular particle was identified, where the EDS analysis confirms the TiB_2_ phase (Spectrum 1, Figure 9). The EDS analysis of the matrix shows aluminum (Spectrum 6, Figure 9).

### 3.2. Optimizing the Inoculant in the AlSi7Mg0.3 Alloy

When using inoculants based on Al-Ti-B type, it is possible to achieve a significant refinement of the α-solid solution in aluminum alloys by adding approximately 0.04–0.05% of Al-Ti-B alloy. Compared to this amount, after increasing the amount of inoculant to 0.1% Al-Ti-B, only a slight refinement of the α-phase occurs, and after exceeding this amount, no further refinement of the a α-phase occurs, as stated in the literature [32]. However, the question of the optimal amount of inoculant for a specific aluminum alloy is not resolved. 

**Figure 10:**
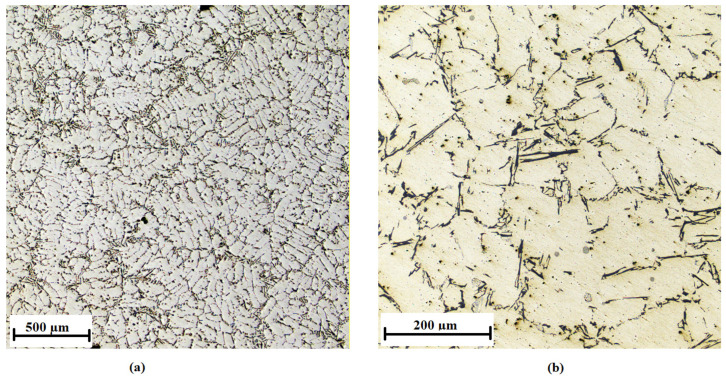
Microstructure of the AlSi7Mg0.3 alloy without inoculants: (**a**) low-magnification overview, (**b**) detail.

**Figure 11:**
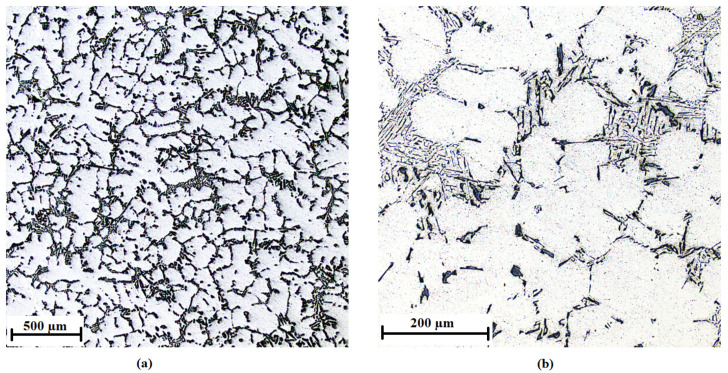
Microstructure of the AlSi7Mg0.3 alloy with the addition of 0.01 wt % AlTi5B1: (**a**) low-magnification overview, (**b**) detail.

**Figure 12:**
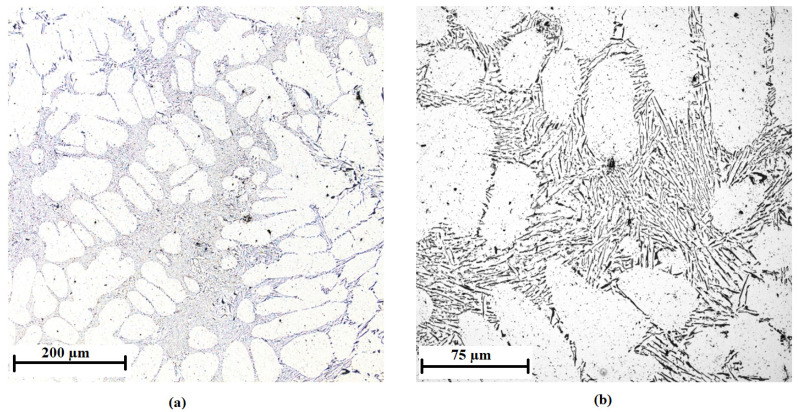
Microstructure of the AlSi7Mg0.3 alloy with the addition of 0.05 wt % AlTi5B1: (**a**) low-magnification overview, (**b**) detail.

**Figure 13:**
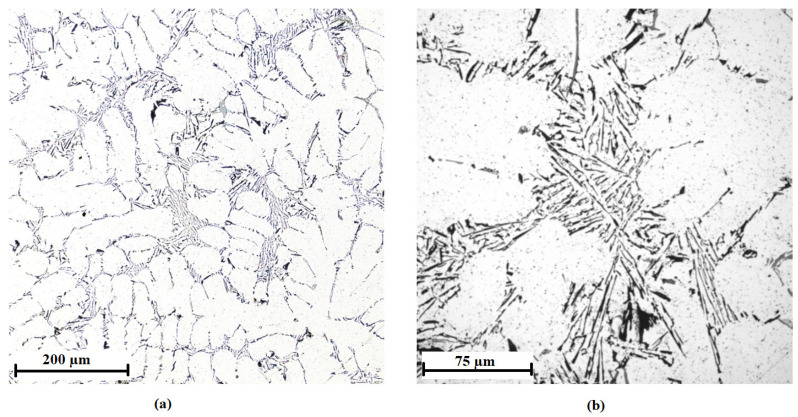
Microstructure of the AlSi7Mg0.3 alloy with the addition of 0.1 wt % AlTi5B1: (**a**) low-magnification overview, (**b**) detail.

**Figure 14:**
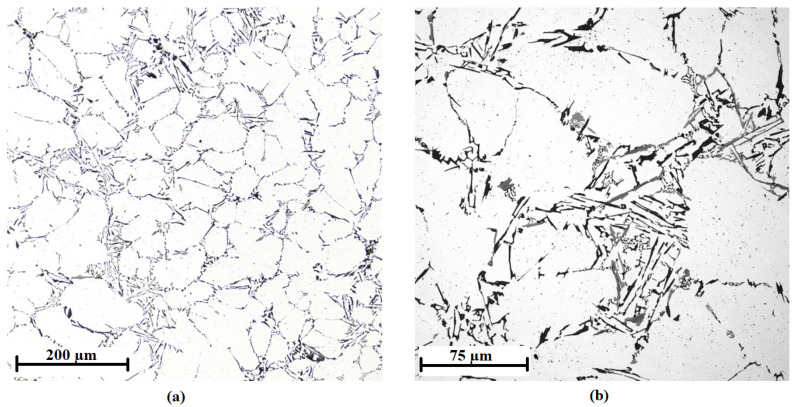
Microstructure of the AlSi7Mg0.3 alloy with the addition of 0.2 wt % AlTi5B1: (**a**) low-magnification overview, (**b**) detail.

The AlSi7Mg0.3 alloy without the addition of inoculants is shown in Figure 10, the addition of 0.01 wt % AlTi5B1 is shown in Figure 11, the addition of 0.05 wt % AlTi5B1 is shown in Figure 12, the addition of 0.1 wt % AlTi5B1 is shown in Figure 13, and the addition of 0.2 wt % AlTi5B1 is shown in Figure 14. 

From all of the micrographs shown, it is evident that the finest dendritic structure is in the samples with an inoculant content of 0.01% (Figure 11). The microstructure consists of an α-solid solution and the eutectic structure. The grains are practically all polyhedral and allotriomorphs. The eutectic is formed by an α-solid solution and silicon in the form of plates that appear as irregular sharp-edged needles of different sizes in the plane of the metallographic cut. In the microstructure with a lower inoculant content (0.05 wt %), the effect of inoculation is noticeable, where the eutectic silicon has a round or slightly elongated shape (Figure 12). In the sample with a higher inoculant content (0.1 wt %, Figure 13), the structure is already different from the previous samples, where the rounded grains of eutectic silicon are slightly elongated. This means that further increasing the content of the inoculant would no longer bring an additional effect of refining the structure of the casting. From a microscopic point of view, one more partial difference can be observed between the content of 0.2 wt % (Figure 14) and 0.1 wt % (Figure 13). At a content of 0.2 wt % inoculant, the α-grains are more polyhedral, and there is a higher area fraction of the eutectic than in the sample with the inoculant content of 0.1 wt %.

In addition to observing the microstructure with a laser microscope, SDAS analysis of individual samples was performed in the AlSi7Mg0.3 alloy without and with various contents of the inoculant. The results are shown in Table 2.

**Figure 15:**
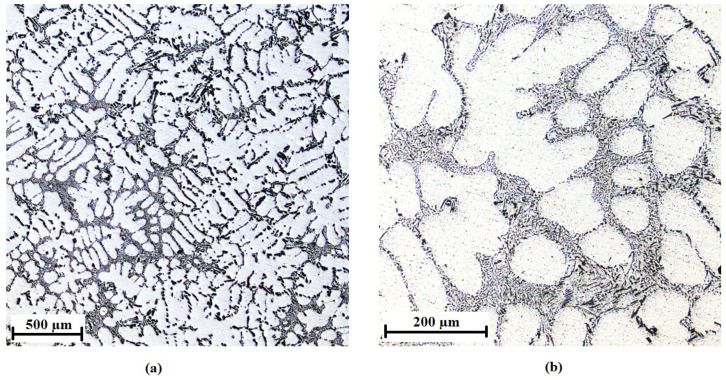
Microstructure of the AlSi7Mg0.3 alloy with the addition of 0.01 wt % AlTi3B1: (**a**) low-magnification overview, (**b**) detail.

The values in the table show that during the crystallization, there was slow cooling, which caused the formation of large grains. The distance of the secondary axes of the dendrite depends on the percentage content of the inoculant and the cooling rate. This means that the larger the SDAS, the coarser the grain and the lower the crystallization nucleus amount. In addition, it can be seen that the addition of inoculant at a concentration of 0.01 wt % caused the greatest increase in the number of crystallization nuclei. On the contrary, the addition of a higher amount of inoculant caused a decrease in the number of crystallization nuclei. The most suitable amount of inoculant appears to be 0.01 wt %. This amount of inoculant was also tested by adding AlTi3B1 to AlSi7Mg0.3 alloy. The microstructure of this alloy is shown in Figure 15. The average value of SDAS in this case is 42 ± 0.3 µm. The difference between the addition of AlTi3B1 and AlTi5B1 is minimal.

## 4. Discussion

From a qualitative point of view, when using the inoculation wire, it is necessary to monitor several structural parameters to achieve the maximum effect of the inoculation. The producer of the inoculation alloys guarantees the chemical composition but not the distribution and size of the particles, which are very important for nucleation. It is mainly the uniform distribution of TiB_2_, (TiV)B_2_, (Al,Ti)B_2_ and AlB_12_ phases without the formation of compact clusters and the size of TiAl_3_ intermetallic phase, max. 40–50 µm without the formation of clusters. The optimal holding time at the inoculation temperature for Al-Ti-B alloys is in the range of 5–10 min [32] (6–7 min were used in this work). Large particles of TiAl_3_ intermetallic phases prolong this time due to their slower dissolution in the melt during inoculation. It should be noted that long-term monitoring and evaluation of the quality of the inoculation wire in terms of the qualitative factors have shown that its quality is variable and there are often cases of noncompliance with these structural parameters. Therefore, it is necessary to regularly evaluate the quality of the inoculation wire in terms of structure prior to inoculation.

**Figure 16:**
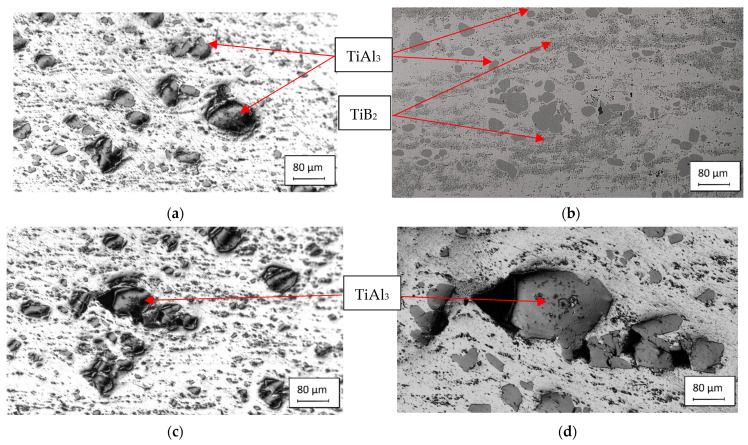
Unsatisfactory microstructure of AlTi5B1 inoculation wires: (**a**) coarse intermetallic phases; (**b**) large compact clusters of intermetallic phases; (**c**) agglomerating intermetallic phases; (**d**) intermetallic phases disintegrated by forming.

The unsatisfactory microstructure of the inoculation wires is shown in Figure 16a, where separate coarse TiAl_3_ intermetallic phases are visible. The coarse intermetallic phases reach a size of 50 to 80 µm and increase the risk of non-dissolution of these particles in the aluminum melt. Figure 16b shows coarse particles of the TiAl_3_ intermetallic phase and very fine particles of TiB_2_, which are not evenly distributed in cross-sections, form large compact clusters, and segregate and degrade during nucleus formation. Another case of an unsatisfactory structure may be the appearance of size-matched but agglomerating TiAl_3_ intermetallic phases (Figure 16c) and/or coarse TiAl_3_ particles disintegrated during forming (Figure 16d) of the inoculation wire. 

## 5. Conclusions

The purpose of this work was to characterize the commonly used AlTi5B1 and AlTi3B1 inoculants to refine the structure of Al-Si alloys. The phase composition of the inoculation wires consists of aluminum, TiAl_3_ titanium aluminide, and TiB_2_ titanium diboride. The microstructure of the inoculant wires is characterized by an aluminum matrix with randomly oriented and shaped titanium aluminide particles, in which titanium diboride particles are located. The average particle size of titanium aluminide is between 37 and 41 µm. Furthermore, the optimal addition of the inoculant to the AlSi7Mg0.3 alloy was investigated. The addition of the inoculant based on titanium is important for the refinement of the casting grains. It was found that the optimal amount of inoculant is 0.01 wt % (the structure was refined by approximately 32%), which causes the greatest refinement of the α-Al grains. The difference between the inoculants AlTi5B1 and AlTi3B1 in grain refinement is minimal. 

## Figures and Tables

**Table 1:** Composition of the alloys.

Number of Sample	Alloy	Amount of the Inoculant Wire AlTi5B1 (wt %)	Amount of the Inoculant Wire AlTi3B1 (wt %)
1	AlSi7Mg0.3	0	0
2	AlSi7Mg0.3	0.01	0
3	AlSi7Mg0.3	0.05	0
4	AlSi7Mg0.3	0.1	0
5	AlSi7Mg0.3	0.2	0
6	AlSi7Mg0.3	0	0.01

**Table 2:** SDAS analysis of individual samples in AlSi7Mg0.3 alloy.

AlTi5B1	Average SDAS (µm)	Standard Deviation (µm)
0 wt % Ti	61	2.08
0.01 wt % Ti	41	0.38
0.05 wt % Ti	45	0.60
0.1 wt % Ti	55	2.42
0.2 wt % Ti	58	3.60

## Data Availability

Data are contained within the article.
